# Diabetes can change the viscoelastic properties of lymphocytes

**DOI:** 10.1007/s40204-018-0096-z

**Published:** 2018-09-01

**Authors:** N. Parvanehpour, Shahrokh Shojaei, S. Khorramymehr, V. Goodarzi, F. Hejazi, V. Faghihi Rezaei

**Affiliations:** 1 Department of Biomedical Engineering, South Tehran Branch, Islamic Azad University, Tehran, Iran; 2 Department of Biomedical Engineering, Central Tehran Branch, Islamic Azad University, Tehran, 13185/768 Iran; 3 Stem cells Research Center, Tissue Engineering and Regenerative Medicine Institute, Central Tehran Branch, Islamic Azad University, Tehran, 13185/768 Iran; 4 Hard Tissue Engineering Research Center, Tissue Engineering and Regenerative Medicine Institute, Central Tehran Branch, Islamic Azad University, Tehran, 13185/768 Iran; 5 Department of Biomedical Engineering, Sciences and Research Branch, Islamic Azad University, Tehran, Iran; 6 Applied Biotechnology Research Center, Baqiyatallah University of Medical Sciences, Tehran, 19945/546 Iran

**Keywords:** Mechanical properties, Viscoelastic, Lymphocytes, Diabetes

## Abstract

Mechanical properties of the cells are among the most highlighted area of interests among researchers for decades. Not only many of the cells’ crucial functional characteristics such as adherence to the cellular substrate, migration abilities and morphological factors are directly influenced by their mechanical properties but also changes in these traits could have importance in diagnosis and even treatments of some serious diseases. The general mechanical properties of the cells are associated with some intercellular characteristics such as arrangement and organization of the actin fibers and cytoskeleton architecture. Any changes due to pathological conditions in the molecular and cellular processes related to these elements can alter the cells’ mechanical characteristics. In this paper, the viscoelastic properties of diabetic and normal lymphocytes were analyzed and compared by application of the iron nanoparticles attached to the cellular membrane and putting the cells in a magnetic field with certain frequency and intensity. Step force was applied to the normal and diabetic lymphocytes and their membrane displacement was tracked by special software and plotted with respect to time. Fitting the experimental data on theoretical formulation of standard linear viscoelastic model, it was demonstrated that diabetic lymphocytes have significantly different viscoelastic characteristics. The results of this paper can be of importance in assessments of diabetic lymphocytes’ abilities to fulfill their immune surveillance tasks.

## Introduction

Cell mechanical properties play pivotal roles in vital characteristics of cells. Many of the biophysical and biological peculiarities are determined by viscoelastic properties of cells (Hayot et al. 2012; Hecht et al. 2015). For instance, it has been illustrated that interaction of a cell and the extracellular matrix is regulated by the cell’s mechanical properties (Trappmann and Chen 2013) or these mechanical traits have significant role in cell signaling (Humphrey et al. 2014). In addition, the cells’ mechanical properties can be regarded as markers of differentiation (González-Cruz et al. 2012; Mathieu and Loboa 2012), pathology (Lekka et al. 2012; Rebelo et al. 2013; Suresh et al. 2015) and transformation (Plodinec et al. 2012). Since different cell sources and different methods such as micropipette aspiration (Zhao et al. 2009), atomic force microscopy (AFM) (Cartagena and Raman 2014; Hecht et al. 2015), magnetic beads microrheometry (Bausch et al. 1998) and others have been utilized for determination of cells’ viscoelastic properties, there is a relative incongruity in results. Therefore, the mechanical properties of cells can be regarded as biomarkers that can be used in diagnosis of some diseases and analyzing the appropriate functioning of cells. In contrast to other methods of measuring viscoelastic properties, the use of magnetic field encompasses the advantage of not having direct contact with the cell body. In the methods such as AFM which include direct contact of an external tip or probe with the same dimension of a cell would lead to active cellular reaction that can easily change the mechanical properties (Guck et al. 2005). In addition, special preparations that are included in some other methods can alter the physiological and biological conditions and lead to results which are significantly different from ordinary homeostatic conditions. Using nanomagnetic adhesive beads accompanied with low-level field seems to have the lowest intervention and, therefore, gives rise to one of the most precise answers. Furthermore, magnetic-oriented approaches seem to be cheaper and simpler in comparison to other methods and so it can be widely and easily used in diagnostic and therapeutic purposes.

Lymphocytes are a small form of leukocytes that can make significant contribution in immune responses. The metabolism and natural biological processes within these cells change due to some diseases such as diabetes (Otton and Curi 2002; Otton and Curi 2002) and, therefore, it is expected to see alteration in membrane mechanical properties in normal and diabetic lymphocytes. In this research, the viscoelastic properties of normal and diabetic lymphocytes were determined and compared by low magnetic field.

## Materials and methods

### Cell culture

The lymphocytes in normal and diabetic groups were provided by Iranian Biological Research Center by ficoll method. RPMI 1640 media contained 10% FBS, 1% penicillin/streptomycin and 1 μg/mL phytohemagglutinin (PHA). The cell suspensions of two groups were incubated separately in culture medium for 18 h, followed by addition of magnetic Fe_3_O_4_ nanoparticles and glucose with certain concentration. The cells of both groups were incubated for another 24 h in CO_2_ incubator for absorption of the magnetic nanoparticles.

### Magnetic field application

**Fig. 1:**
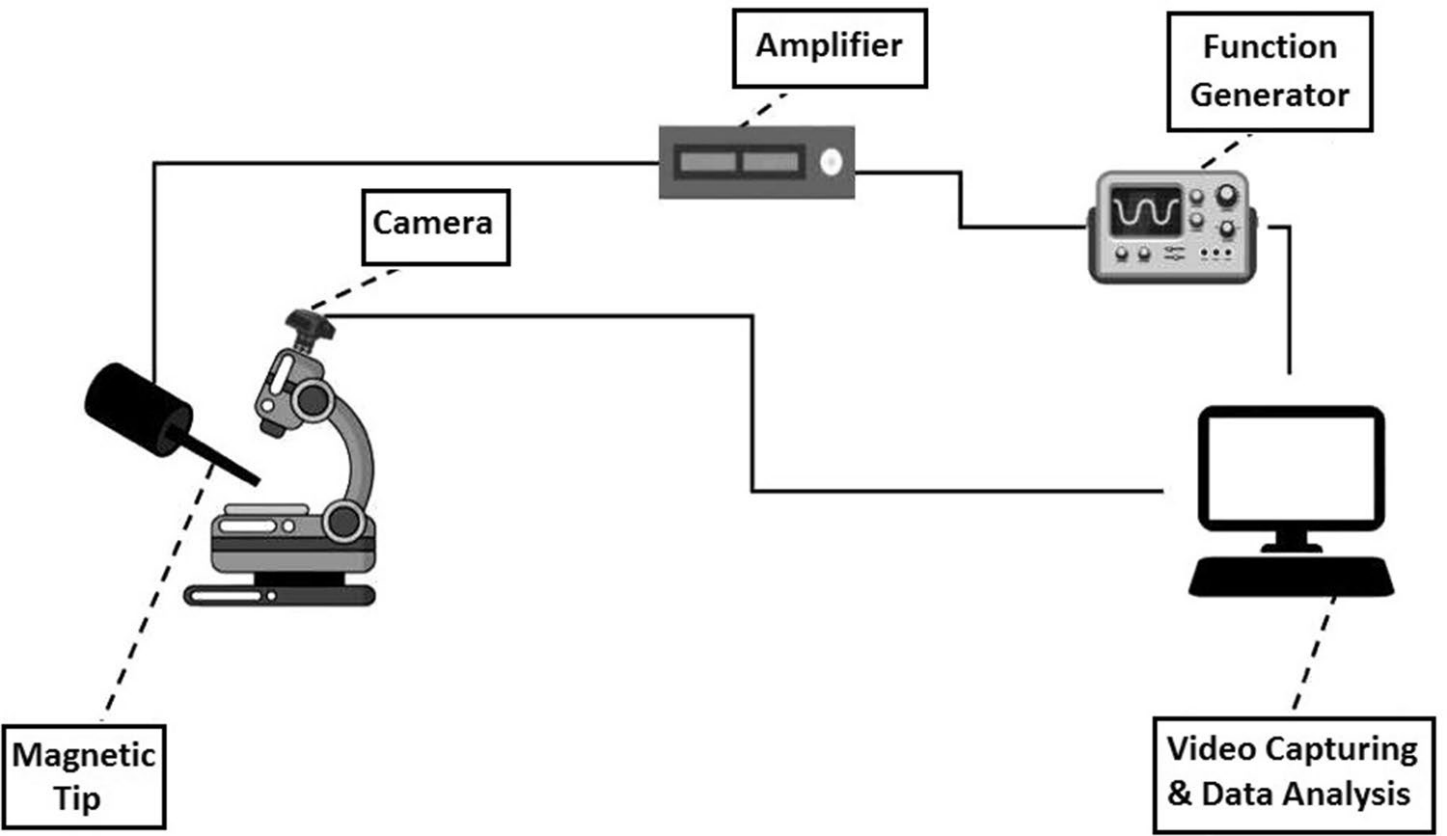
The schematic view of the system and its elements’ arrangement

Since the lymphocytes are characterized as non-adhesive cell types, for test one droplet of the cell suspension was applied to the laboratory neubauer lam. Cells were transferred on neubauer lam and their displacements were recorded after application of the magnetic field under the optical microscope (1600×). For each test, first a single cell has been located and different magnitudes of forces were applied under the effect of different magnetic fields. The displacement of cells under the effect of forces was recorded by a 2 mega pixels 60 frames per second camera. Figure 1 shows the schematic view of the inductance system. A teslameter device (Pazhoohesh Nasir Model 1394) was used for assessing the intensity of the magnetic system. This teslameter can sense the intensity of the magnetic field in three Cartesian coordinates with the accuracy of 0.001. The cells were subjected to 840 µT magnetic field of 0.2 sq.Hz.

### Magnetic inductance system

With the use of a function generator, the desired wave form could be produced and after amplification to 4 A the electrical current was applied to a coil. Magnetic field has the effects on magnetic nanoparticles which are connected to cell membrane. The coil has two cores. The central iron core has the radius of 2.5 cm and the length of 7 cm and the secondary Si core has the length of 2.5 cm and wrapped around the central core. The copper wire of 1.1 mm was twisted 400 times around the cores. A teslameter with the accuracy of 0.001 was located in an appropriate location for determination of the magnitude of magnetic field.

### Date processing

The videos are transferred into computer for processing. On the computer, four specific softwares for this purpose have been installed. The function generator software induces the desired wave form to the generator, Teslameter software that shows the magnitude of the magnetic field in micro Tesla in three Cartesian directions, video recording software that is related to the microscope and finally the software of Tracker (version 4.81) analyzing the displacement.

### Viscoelastic model

**Fig. 2:**
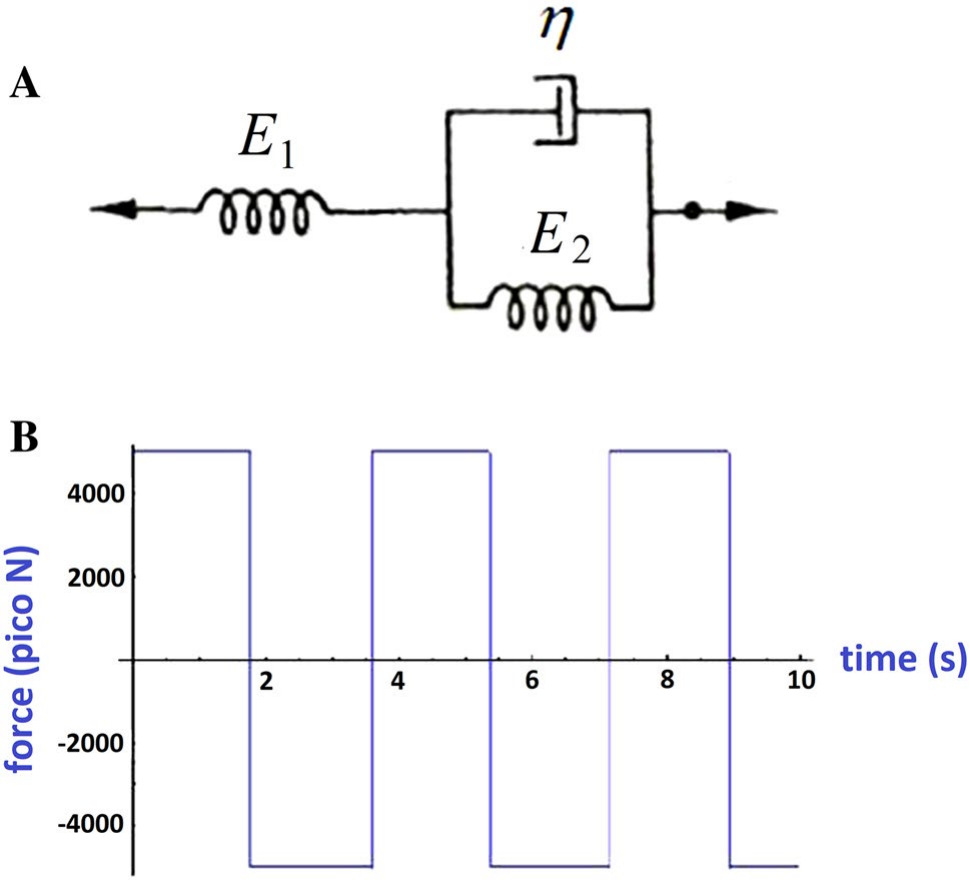
**a** The standard linear model for viscoelastic materials. This model consists of two springs and a dashpot. **b** The applied square shape force with time. The samples were exposed for 15 s

Standard linear model was considered for force and displacement relation. This model consists of a spring and a Kelvin model in series. This model is shown in Fig. 2a.

The relation between stress and strain can be readily obtained as below (Eq. 1). This model has been widely used in previous biological researches (Lim et al. 2006).

1 $$ \sigma (t) + \frac{{\frac{\eta }{{E_{1} }}}}{{1 + \frac{{E_{2} }}{{E_{1} }}}}\dot{\sigma }(t) = \frac{{E_{2} }}{{1 + \frac{{E_{2} }}{{E_{1} }}}}\varepsilon (t) + \frac{\eta }{{1 + \frac{{E_{2} }}{{E_{1} }}}}\dot{\varepsilon }(t) $$

By substituting the coefficients with *p*_1_, *q*_0_ and *q*_1_ we have Eq. 2.

2 $$ \sigma (t) + p_{1} \dot{\sigma }(t) = q_{0} \varepsilon (t) + q_{1} \dot{\varepsilon }(t) $$

And by applying the step function with amplitude of *σ*_0_ for the *σ* function we will have Eq. 3.

$$ \sigma (t) = \sigma_{0} H(t) $$

3 $$ \to \varepsilon (t) = \frac{{\sigma_{0} }}{{q_{1} }}\left[ {\frac{1}{\lambda }\left( {1 - e^{ - \lambda t} } \right) + p_{1} e^{ - \lambda t} } \right];\;\lambda = \frac{{q_{0} }}{{q_{1} }} $$

The parameters *p*_1_, *q*_0_ and *q*_1_ should be determined in practical experiment. Figure 2b shows the applied force. The frequency was 0.2 Hz, 4 A electrical current and the magnetic field 840 μT. The samples were exposed to this field for 15 s.

### Statistical analysis

All the tests were performed three times and in each test at least ten cells from each group were selected. *T* test-paired statistical analysis was done for the three constants of the Eq. 3 and *P* value below 0.05 was set as the criterion of significant difference.

## Results

**Fig. 3:**
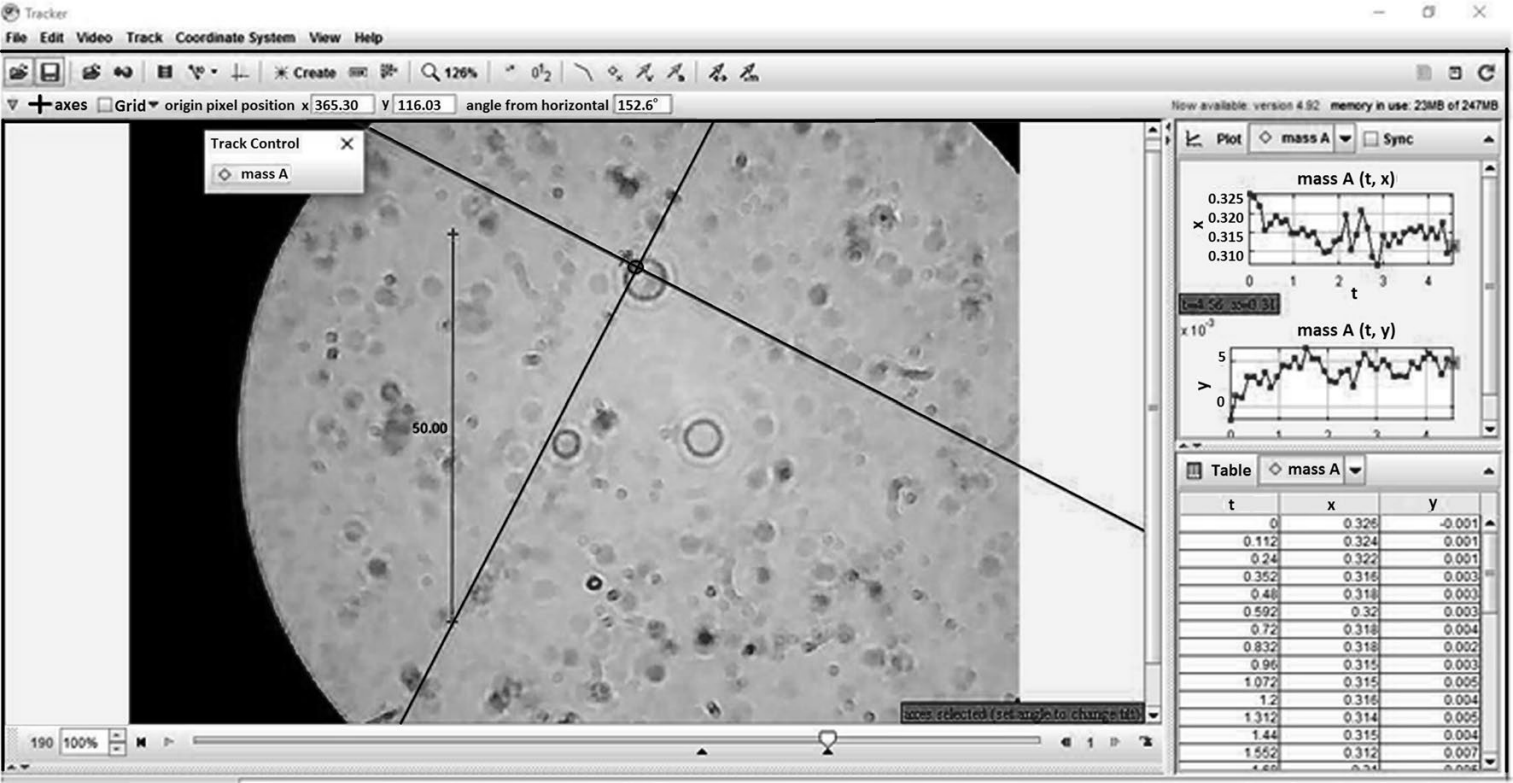
The displacement of the cell membrane in *x* and *y* directions. Ten random cells were selected and the final result is the average of displacement in each group

The displacement of the cell membrane in different normal and diabetic groups was analyzed after application of magnetic field. Figure 3 shows the medium of the Tracker software which includes the one specific cell. The *x*- and *y*-direction displacements with time for the membrane have been depicted in Fig. 3.

**Fig. 4:**
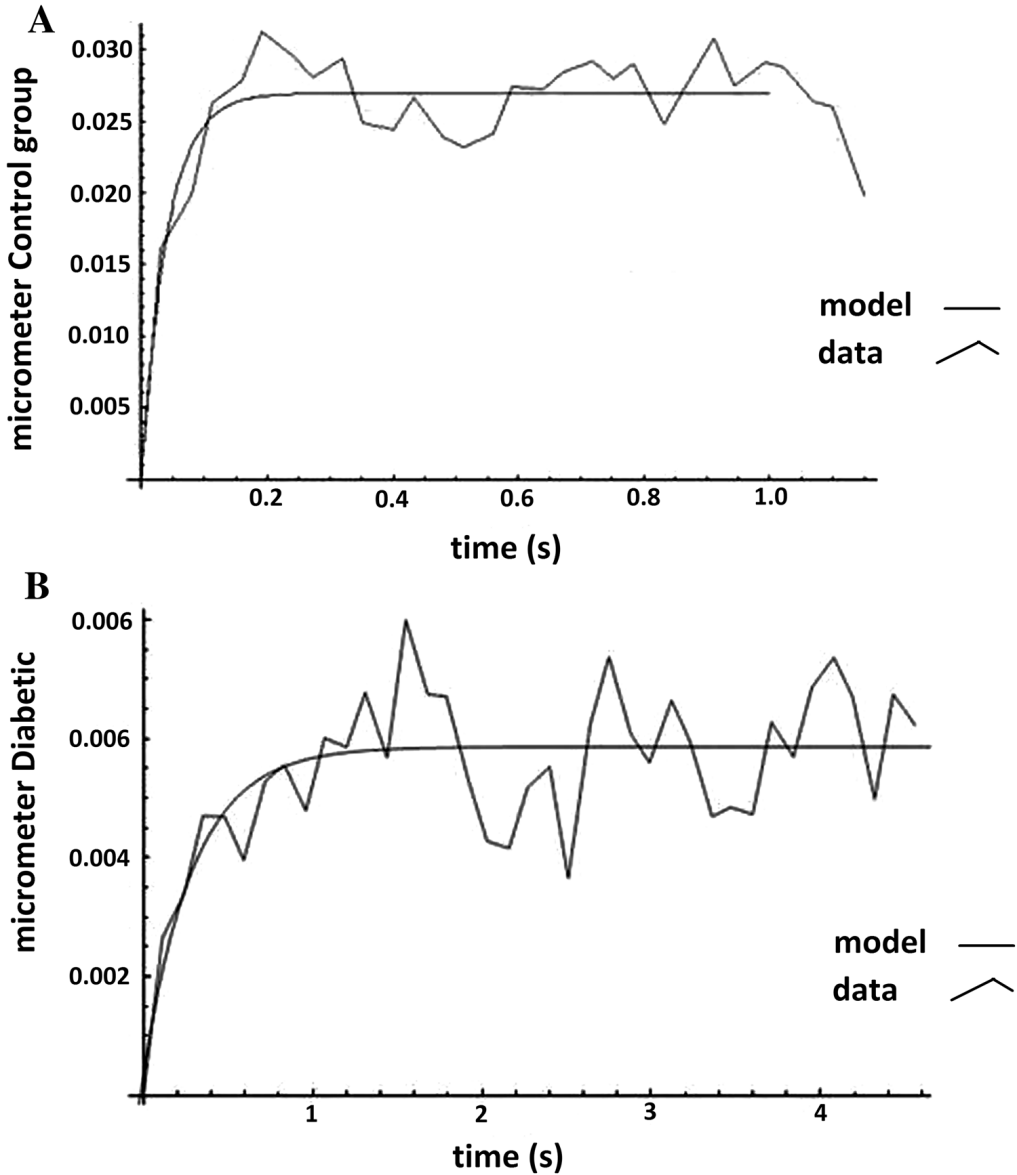
Membrane displacement with respect to the time after application of magnetic field for normal (**a**) and diabetic (**b**) lymphocytes. In both graphs the experimental data have been depicted together with theoretical data derived by the standard viscoelastic model

Figure 4 shows the experimental and theoretical displacement variations with respect to time. Experimental points have been plotted and connected together and the theoretical curve was fitted by MATLAB software version (version 7.3) to the experimental data by obtaining the most appropriate constant values of Eq. 3. Figure 4a depicts the experimental and theoretical displacement curves with respect to time for normal lymphocyte and Fig. 4b is related to the diabetic lymphocyte.

Table 1 shows the constant values of Eq. 3 for normal and diabetic lymphocytes based on the fitted curve to the experimental data.

**Table 1:** The constants of the standard linear model of viscoelastic material for normal and diabetic lymphocytes

Constants of Eq. 3	Normal lymphocytes	Diabetic lymphocytes	*P* value
*p*_1_	0.0074 ± 0.0008	0.2522 ± 0.0036	≤ 0.05
*q*_0_	0.1852 ± 0.03145	0.8528 ± 0.0752	≤ 0.05
*q*_1_	0.0003 ± 4.1 × 10^−5^	0.0175 ± 0.00277	≤ 0.05

## Discussion

The mechanical properties of cells and tissues have attracted many scientists’ and researchers’ attention. It has been demonstrated that changes in mechanical characteristics of cell can be one of the best criteria in early diagnosis of many diseases. In addition, cellular well-functioning is attributed directly to mechanical properties of those cells. Due to these facts, recently some papers have been published on measuring viscoelastic properties of lymphocytes. To the best of our knowledge, this is the first time that the viscoelastic properties of normal and diabetic lymphocytes were measured and analyzed by this approach. While many researchers use micropipette aspiration or AFM method, in this research the viscoelastic properties of normal and diabetic lymphocytes have been investigated by application of magnetic field on iron nanoparticles-loaded cells. In this research, the viscoelastic properties of normal and diabetic lymphocytes were assessed and obtained. Magnetic iron nanoparticles have been added to the culture medium of the lymphocytes with certain concentration and by application of magnetic field in the graph of membrane displacement with respect to the time was plotted. By utilization of standard linear viscoelastic model, the mechanical properties of normal and diabetic lymphocytes were investigated and compared.

The results demonstrated that diabetes can change the mechanical properties of lymphocytes. Any physical (Rebelo et al. 2013; Rianna and Radmacher 2016) and chemical (Peetla et al. 2013) alterations at the cells’ surface can readily lead to intervention in cells’ vital functions, malfunction of entering and exit process of the necessary chemicals and even disorder in secretion of enzymes, proteins and other substances. As the previous researches have illustrated that diabetes can change some pivotal traits of lymphocytes such as metabolism rate (Otton and Curi 2002) or apoptosis (Otton et al. 2004), and also because of the interconnected physical and functional characteristics of the cells it was expected to see different mechanical properties for normal and diabetic lymphocytes. Our results have proven this hypothesis.

There are evidences that demonstrate the close relationship between the mechanical properties of cells’ membrane and the organization and arrangement of their actin cytoskeleton (Lekka et al. 2011, 2012). It means that any change in actin fibers’ arrangement and organization will cause change in mechanical properties of the whole cell. In addition, there are sufficient clues which illustrate the direct connection between the actin cytoskeleton remodeling properties and the cell motility. Past research works have shown that the remodeling of the dynamic filament meshwork is one of the primary influential factors in the cells’ migration abilities (Gardel et al. 2010; Ridley et al. 2003). Therefore, the mechanical characteristics of cells including viscoelastic properties can be regarded as one of the essential parameters which depict the migration capability. This issue even becomes more highlighted for lymphocytes as their immune surveillance tasks are closely interconnected with their motile abilities (Dupré et al. 2015).

The results of this paper show a significant difference between the viscoelastic properties of normal and diabetic lymphocytes. Based on the previous discussion, this means a discrepancy exists between some vitally important characteristics of normal and diabetic lymphocytes such as their migration ability and, therefore, it can jeopardize diabetic lymphocytes’ efficiency in immune responses.

By the hypothesis of regarding the architecture of the cytoskeleton as one the primary influential factors in total mechanical properties of a cell, we can associate the changes in viscoelastic properties of diabetic lymphocytes to the molecular and chemical mechanism which control the structure of cytoskeleton. Many proteins such as the family of ERM (ezrin, radixin, moesin) which contribute as actors in remodeling of cytoskeleton (Chen et al. 2013) can change in diabetic situation (Nishida et al. 2014).

## Conclusion

In this research, the viscoelastic properties of normal and diabetic lymphocytes have been investigated and significant differences have been identified. It has been understood that the mechanical properties of healthy and diseased lymphocytes are different in terms of their energy storing and dissipating rates. While cellular migration includes continuous membrane bending and deformation, it can be deduced that the diabetic cells have different migration pattern. Finally, while the most important function of the lymphocytes necessitates their facilitated movement, diabetic cells seem to have problems in performing their tasks.
